# Choroidal vascular changes in retinitis pigmentosa patients detected by optical coherence tomography angiography

**DOI:** 10.1186/s12886-020-01640-5

**Published:** 2020-09-29

**Authors:** Chang Shen, Yang Li, Qian Wang, Yu-Ning Chen, Wei Li, Wen-Bin Wei

**Affiliations:** 1 Beijing Tongren Eye Center, Beijing Tongren Hospital, Beijing Ophthalmology and Visual Science Key Lab, Beijing Key Laboratory of Intraocular Tumor Diagnosis and Treatment, Capital Medical University, Beijing, China; 2 State Key Laboratory of Stem Cell and Reproductive Biology, Institute of Zoology, Chinese Academy of Sciences, Beijing, 100101 China; 3 University of Chinese Academy of Sciences, Beijing, 100049 China

**Keywords:** Choroidal vascularity, Choriocapillaris;large-angle OCT angiography, Retinitis pigmentosa, Choroidal vascularity index

## Abstract

**Background:**

To evaluate choroidal vascular changes, including choriocapillaris (CC) and middle/large choroidal vessels, in retinitis pigmentosa (RP) patients using wide-angle optical coherence tomography angiography (OCTA) and to determine whether changes in the choroidal vascularity have a relationship with visual function and retinal structural changes.

**Methods:**

34 patients with a confirmed diagnosis of RP and 48 controls were recruited. All patients underwent detailed ophthalmologic and imaging examinations, including two types of OCTA (Optovue, 3 × 3 mm, 6 × 6 mm; VG-200, 12 × 12 mm). CC defects were defined according to the choroidal vascular structure in five degrees. To evaluate middle and large choroidal vascular changes, the choroidal vascularity index (CVI), which was the luminance volume to the total choroidal volume, was used.

**Results:**

Defects of choroidal vascularity of RP eyes were detected in comparison to control eyes. The defects were observed in the CC layer with a concentric or lobular pattern at different degrees. CVI, which was used to reflect middle/large choroidal vascularity, decreased in the perifoveal, pararetinal and periretinal regions in the RP eyes. CC defects degree were correlated with the BCVA (*p* = 0.001, r = − 0.556), the Humphrey indexes (mean deviation, MD, *p* < 0.001, r = − 0.673; PVF, *p* = 0.003, r = − 0.639; 10° mean sensitivity, 10° MS, *p* = 0.002, r = − 0.651) and microperimetry index (mean sensitivity, MS, *p* < 0.001, r = − 0.807). The preserved CC area (mean value: 28.65 ± 12.50 mm^2^) was negatively correlated with MS measured by microperimetry (*p* = 0.005, r = − 0.449). Ordinary regression analysis revealed that the CC defect degree was associated with the CVI of perifovea (*p* = 0.002, 95% CI: − 102.14 to − 24.01), the EZ length (*p* = 0.006, 95% CI: − 0.006 to − 0.001) and the VAD (vascular area density) of the DCP (deep capillary plexus) in the fovea (*p* = 0.022, 95% CI: 0.038–0.478). No correlations were detected between BCVA and CVI in any retinal regions. No correlations were found between the CVI and the VAD in any retinal regions.

**Conclusion:**

The choroidal vascularity was widely defected in RP. Choriocapillaris and middle/large choroidal vascularity defects were correlated with each other. Visual function and retinal structural changes were found to be associated with choriocapillaris defects but not with middle/large choroidal vascular defects.

## Background

Retinitis pigmentosa (RP) is a group of progressive degenerative eye diseases with a prevalence of 1/4000 worldwide [1]. RP is characterized by progressive degeneration of the visual cells and abnormalities in the retinal pigment epithelium (RPE), with an outcome of blindness typically achieved over a period of several decades, during which vision diminishes slowly and visual function is completely lost in the late stage.

Choroidal vascularity, which provides nourishment, oxygen supply and metabolism exchange to the outer retina and RPE, plays an important role in RPE atrophic diseases. Swept-source OCT angiography (SS-OCTA) with a light source of 1050 nm can provide detailed and accurate choroidal images [2]. CC defects have been shown to have a significant association with RPE and photoreceptor atrophy, which have been reported in geographic atrophy [3], drusen [4] and diabetic retinopathy [5]. In RP, small angle OCTA (3 mm × 3 mm or 6 mm × 6 mm) failed to detect differences in CC in comparison to healthy controls [5]. Since the peripheral retina is more easily affected in RP, large-range SS-OCTA is needed. Recent studies using 12 × 12 mm or 8 × 8 mm OCTA have found choriocapillaris flow voids or flow defects in certain regions of the retina [6, 7], but the relationship between CC defects and retinal or choroidal structural changes has never been reported.

The choroidal vascularity index (CVI) is a novel OCT-based choroidal vascularity marker that reflects the relationship between the choroidal luminance volume and the total choroidal volume. CVI has been used to represent the choroidal vessel condition in central serous chorioretinopathy [8]. Previous studies on retinal dystrophies have revealed a decrease of CVI in comparison to that of normal controls [9]. However, these studies all focused on one B-scan passing through the fovea but did not investigate the perifoveal region, which would be more likely to be affected in RP eyes. Recently, a method of evaluating the three-dimensional CVI was reported [2], which can be used to evaluate the middle and large choroidal vessels in a cubical pattern. Focusing on specific regions of the three-dimensional CVI map may help to understand the middle and large choroidal vascular changes in RP.

The aim of this study was to characterize choroidal circulation changes, including choriocapillaris and middle/large choroidal vessels by using two types of OCTA, and to determine whether changes in the choriocapillaris and middle/large choroidal vessels have a relationship with visual function and retinal structural changes.

## Methods

### Study population

The present study was a prospective observational cohort of 34 patients who were recruited from the Beijing Tongren Eye Centre with a diagnosis of RP from January 2019 to October 2019. For retinal evaluation, 32 normal control individuals were recruited from the Kailuan Eye Study as the control group, who were age- and gender-matched with the RP patients [10]. For choroidal evaluation, 17 normal control eyes from patients were recruited from the outpatient department in Beijing Tongren Eye Centre who underwent VG-200 OCTA examination and other ophthalmic examinations.

The study was approved by the national research ethics committee, and all participants gave informed consent to participate. All study procedures followed the Declaration of Helsinki.

### RP diagnosis and enrollment inclusion and exclusion criteria

The diagnosis of RP was confirmed by an experienced ophthalmologist (Dr. Wei Wenbin) with the following diagnosis criteria: 1) typical history including night blindness and vision loss; typical fundus appearance including bone spicule-like pigmentation, constricted retinal arteries and pale optic nerve head; 2) presence or absence of a family history of night-blindness or low-vision; 3) visual field loss detected by static perimetry with ring scotoma or constricted visual fields; and 4) reduced or unrecordable amplitude detected by electroretinogram.

The exclusion criteria for patients included 1) the presence of any retinal disease other than RP; 2) patients with the BCVA (better eye) lower than 0.05 which constricted the the acquisition of OCT or OCTA scans; 3) patients with low cooperation which constricted the acquisition of OCT or OCTA scans; 4) severe cataract, which affected the image quality of OCT or OCTA scans.

For the control group, patients with no history of retinal disease or retinal surgery were included. Patients with cataracts that affected image quality were excluded. Patients with large refractive error (> − 6 D or > + 6 D) were excluded.

### Ophthalmic examinations

All RP patients received comprehensive ocular examinations, including best-corrected visual acuity (BCVA), intraocular pressure (IOP) measurement, slit-lamp biomicroscopy, color fundus photography (Optos 200Tx, Optos, Dunfermline, UK; TRC RETINAL CAMERA 50 DX, Topcon Inc., Tokyo, Japan), ocular biometry applying optical low-coherence reflectometry (Lenstar 900 Optical Biometer; Haag-Streit, Koeniz, Switzerland), two types of OCT angiography (RTVue XR Avanti device, Optovue Inc., Fremont California, USA; VG200, SVision Imaging, Ltd., Luoyang, China), perimetry tests (HFA; Carl Zeiss Meditec, Inc. Dublin, CA, USA) and microperimetry (MP-3, Nidek Technologies, Padua, Italy).

The visual field testing implemented in this study included automated static perimetry tests and microperimetry. The automated static perimetry tests were performed using the central 24–2 Swedish Interactive Thresholding Algorithm Standard Program. The lens was corrected to be appropriate for the test distance. Visual field testing was repeated if the test reliability was not satisfactory (i.e., fixation loss > 20%, false-positive rate > 15% or false-negative rate > 33%). Microperimetry was performed for all patients with a standardized grid of 45 Goldmann III stimuli covering the central 12° using a 4–2 fast strategy.

OCT was performed with the Optovue device using radial and raster patterns. Two types of OCTA were used in this study. Indicator light which can help low-vision patients fixing by the fellow eye and eye-tracking system which was built in the software were used to acquire relatively good quality images from low-vision patients. For retinal vascularity assessments, 3 mm × 3 mm and 6 mm × 6 mm OCTA images captured by RTVue Angio Retina mode were used. The technique including the SSADA method has been described in detail [7, 11]. A total of 304 × 304 scans were captured in a macular cube pattern. For choroidal vascularity assessments, 12 mm × 12 mm SS-OCTA images were captured with the commercial VG200 SS-OCTA device. This device used an SS laser with an approximate wavelength of 1050 nm. Detailed information on the acquisition protocols for this device has been previously reported [2]. 12 × 12 mm OCTA data centered on the fovea were obtained with 1024 × 1024 B-scans.

The control eyes underwent a simplified ocular examination. Controls from the Kailuan Eye Study received visual acuity (VA), IOP measurement, fundus photograph, ocular biometry applying optical low-coherence reflectometry, OCT (Optovue) and OCTA (Optovue, 3 mm × 3 mm, 6 mm × 6 mm) examinations. Controls from the outpatient department of the Tongren Eye Centre received VA, IOP measurement, fundus photography and OCTA (VG-200, 12 mm × 12 mm).

### Visual field measurement procedures

Three indices were derived from the Humphrey test results: 1) the mean deviation (MD) for assessing the whole retina sensitivity; 2) the preserved visual field (PVF), which was defined as the longest diameter with a retinal sensitivity> 0 dB; and 3) the 10° mean sensitivity (10° MS), which was calculated as the mean value of the retinal sensitivity in a field with a diameter of 10°.

For microperimetry analysis, the fixation pattern was regarded as stable if > 75% of the fixation points were inside a 2° diameter circle, as relatively unstable if < 75% were inside the 2° diameter circle but > 75% were inside a 4° diameter circle and as unstable if < 75% of the fixation points were inside the 4° diameter circle [12].

### OCT and OCT angiography measurement procedures

For OCT analysis, the central foveal thickness (CFT, nm), subfoveal choroidal thickness (SFCT, nm) and ellipsoid zone length (EZ length, nm) were measured with built-in software (ReVue, Software Version, 2017,1,0,155). The CFT and SFCT were measured twice in the vertical and horizontal meridian images from radial scans, and the average value was used in the data processing, while the EZ length was measured in the middle image of raster scans.

The vascular area densities (VADs) of the fovea, parafovea, and perifovea acquired through 6 mm × 6 mm OCTA images by ReVue were used in the data processing. The quality index was checked in each examination report, and eyes with a QI lower than 5 (median value) were excluded from the study. Retinal segmentation was checked, and manual manipulation was performed in images that researchers considered unreliable with the user-guided segmentation process built into the software.

For choroidal vascularity assessments, segmentation of the Bruch membrane and the lower choroid boundary were corrected manually using built-in software. Choriocapillary and three-dimensional CVI images were used. Images with patches of deficiency, blurred, or reversal regions were considered as having a low quality and therefore excluded from our research.

### Morphologic description and analysis of Choroidal vessels

**Fig. 1:**
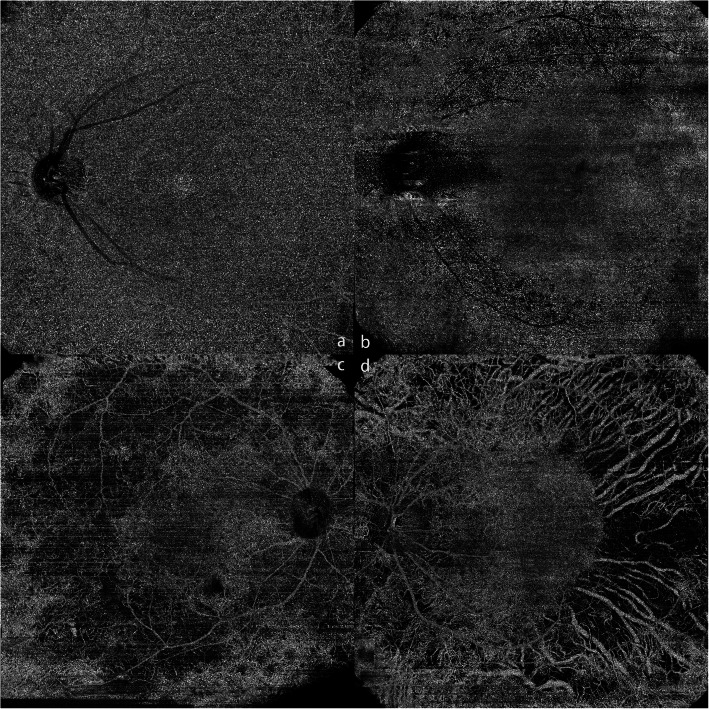
Different degrees of CC defects. a, level a-CC with no defects from a normal control eye; b, level b-areas of decreased density in the choriocapillaris in a lobular pattern; c, level c-areas of exposed, small choroidal vessels; d, level d-areas of exposed, large choroidal vessels

Choriocapillaris angiography acquired from 12 mm × 12 mm SS-OCTA was used for evaluation. Four types of choriocapillaris defects were defined according to the choroidal vascular structure [13] (Fig. 1): a-no defects; b- areas of decreased density in the choriocapillaris; c- areas of exposed, small choroidal vessels detected; d- areas of exposed, large choroidal vessels detected. To detect the relationship between the CC defect degree and visual function, we defined 5 scores for CC defect degrees: 0 = a; 1 = b; 2 = c; 3 = b + c; 4 = c + d; 5 = d.

**Fig. 2:**
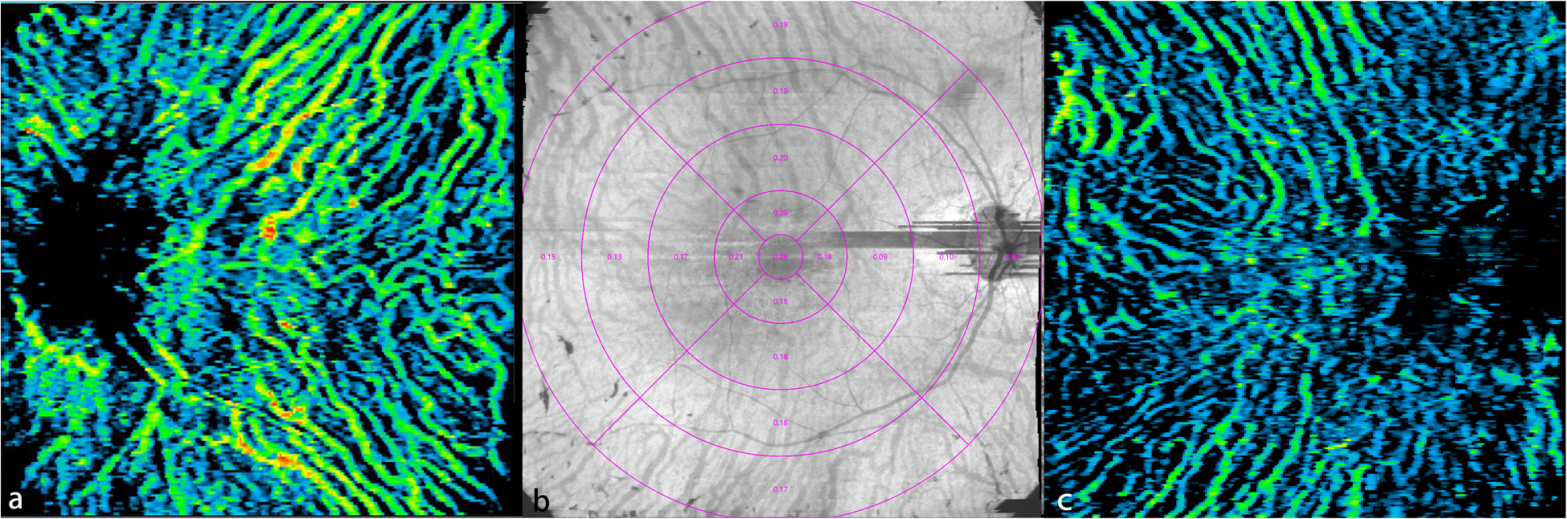
Three-dimensional choroidal vascularity index (CVI) maps: a, CVI color-coded A-scan map from a normal control eye; b, numeric CVI ETDRS map from an RP patient; c, CVI color-coded A-scan map from the same RP patient

The three-dimensional CVI was defined as the ratio of the choroidal vascular luminal volume to the total choroidal volume and was calculated automatically using built-in software in the SS-OCTA device with an ETDRS pattern (Fig. 2).

### Statistical analysis

All analyses were conducted using SPSS (IBM SPSS for Windows, version 23) and GraphPad PRISM version 8.0 (GraphPad Software Inc.) statistical software. Descriptions of the quantitative data are presented as the means (standard deviations, SDs), medians, minimum (Mins) and maximum (Maxs). Categorical data included the number of cases and percentages. All statistical tests used a two-sided test with corresponding 95% confidence intervals (CIs), and *P* < 0.05 was considered statistically significant.

## Results

### Basic characteristics

A total of 34 patients (63 eyes) with a diagnosis of RP were recruited. Among the patients, 52.9% had blurred vision or night blindness from early childhood (< 10 years old), 11.8% from puberty (10s–20s), 26.5% from youth (20s–40s), and 8.8% from middle age (> 40 years old). The study also recruited 2 groups of healthy control individuals who were age- and gender-matched to the RP patients. Group 1 was recruited from the Kailuan Eye study and included a total of 32 individuals. Group 2 was recruited from the outpatient department of Beijing Tongren Eye Centre and included a total of 17 individuals. All basic characteristics of the RP patients are recorded in Table 1. No significant difference was detected between the RP and control groups in terms of age (vs control 1, *p* = 0.381; vs control 2, *p* = 0.779), gender (vs control 1, *p* = 0.794; vs control 2, p = 0.381) or axial length (AXL) (vs control 1, *p* = 0.305).

**Table 1:** Basic characteristics of RP patients

Variables	Mean ± SD
male/female	17/17
age	39.5 ± 14.03
VA	0.36 ± 0.31
IOP, mmHg	12.94 ± 2.73
RE, DS	−2.63 ± 4.02
RE, DC	−0.37 ± 1.53
CFT,μm	173.56 ± 72.89
EZ length,μm	865.19 ± 933.55
SFCT,μm	210.59 ± 93.70

RP, retinitis pigmentosa; VA, visual acuity; IOP, intraocular pressure; RE, refractory error; DS, diopter sphere; DC, diopter column; AXL, axial length;CFT, central fovea thickness; EZ, ellipsoid zone; SFCT, subfovea choroidal thickness

### Retinal and choroidal vascularity assessments

According to the quality control criteria, 46 RP eyes and 31 healthy control eyes were used in retinal vascularity evaluation, while 31 RP eyes and 17 control eyes were used in choroidal vascularity evaluation.

#### Retinal vascularity assessments

Significant decreases were detected between the RP and control groups in terms of SCP and DCP (Table 2).

**Table 2:** OCTA Vessel Density in RP and Control groups

	SCP			DCP		
	Control	RP	*P* value	Control	RP	***P*** value
Fovea	18.81 ± 6.18	13.88 ± 11.5	**0.009**	34.06 ± 6.29	30.58 ± 10.27	0.187
ParaFovea	50.68 ± 3.83	39.63 ± 6.01	**< 0.001**	55.03 ± 3.32	48.1 ± 5.17	**< 0.001**
ParaT	50.17 ± 4.02	41.17 ± 8.98	**< 0.001**	56.02 ± 3.22	48.22 ± 5.93	**< 0.001**
ParaS	52.02 ± 4.7	39.45 ± 7.4	**< 0.001**	54.78 ± 3.59	48.62 ± 5.85	**< 0.001**
ParaN	49.93 ± 4.09	37.27 ± 8.44	**< 0.001**	55.58 ± 3.56	47.6 ± 5.52	**< 0.001**
ParaI	50.59 ± 4.79	40.32 ± 7.54	**< 0.001**	53.74 ± 4.51	47.99 ± 6.48	**< 0.001**
PeriFovea	50.08 ± 2.47	46.65 ± 4.21	**< 0.001**	51.23 ± 5.11	45.57 ± 5.52	**< 0.001**
PeriT	45.02 ± 3.84	41.41 ± 5.49	**0.002**	53.11 ± 5.18	42.72 ± 7.13	**< 0.001**
PeriS	51.14 ± 2.91	48.22 ± 4.92	**0.002**	52.01 ± 6.19	46.21 ± 5.84	**< 0.001**
PeriN	54.34 ± 2.05	50.32 ± 4.31	**< 0.001**	50.45 ± 5.29	48.12 ± 5.95	0.164
PeriI	49.78 ± 2.64	47.19 ± 5.19	**0.007**	49.37 ± 6	45.42 ± 5.77	**0.008**

SCP, superficial capillary plexus; DCP, deep capillary plexus; T, temporal; S, superior; N, nasal; I, inferior

There was no difference found between the control and RP groups in regard to the FAZ area (*p* = 0.935) and perimeter (*p* = 0.444), while the acircularity index increased in the RP group (*p* < 0.001) (Supplement Table 1).

Correlation analysis was performed between the functional indices and retinal vascularity indices, as well as between the OCT measurements and functional indexes (Table 3).

**Table 3:** Correlation analysis between retinal vascularity indexes and functional indexes

		VAD in SCP			VAD in DCP			CFT (um)	EZ length (um)
		Fovea	ParaFovea	PeriFovea	Fovea	ParaFovea	PeriFovea		
BCVA	Pearson r	0.270	−0.230	0.012	0.504	0.223	0.078	0.544	0.545
	*p* value	0.07	0.124	0.936	**< 0.001**	0.136	0.615	**< 0.001**	**< 0.001**
MS, dB	Pearson r	0.159	−0.085	0.146	0.195	0.259	0.246	0.408	0.678
	*p* value	0.292	0.576	0.345	0.195	0.082	0.107	**0.012**	**< 0.001**
PVF, °	Pearson r	0.373	−0.002	0.070	0.241	0.042	0.030	0.371	0.552
	*p* value	**0.028**	0.991	0.694	0.163	0.811	0.867	**0.040**	**0.002**

VAD, vascular area density; SCP, superficial capillary plexus; DCP, deep capillary plexus; BCVA, best-corrected visual acuity; MS, mean sensitivity; PVF, preserved visual field;CFT, central fovea thickness; EZ, ellipsoid zone

Linear regression analysis revealed that the VAD in the DCP of the fovea and the EZ length was associated with the BCVA (*p* = 0.029, *p* = 0.005; standardized coefficients, 0.327, 0.43), while age and axial length were not (*p* = 0.525, *p* = 0.065).

#### Choroidal vascularity assessments

After removal of images with patches of deficiency, blurred or reversal, 17 three dimensional CVI images were included after image quality control. A decrease in the CVI was detected in the RP patients compared to that of the controls in the perifoveal, pararetinal and periretinal regions (Table 4). No correlations were found between the CVI of any region and the BCVA (Table 4). No correlations were found between the CVI of the choroid vascularity and the VAD of the retinal vascularity (Supplement Table 2).

**Table 4:** CVI of RP and control eyes and the correlation analysis with BCVA

	RP	control	*p* value vs control	*p* value of the Pearson test with BCVA
CVI fovea	0.28 ± 0.08	0.28 ± 0.08	0.918	0.099
CVI parafovea	0.25 ± 0.04	0.28 ± 0.06	0.113	0.201
CVI perifovea	0.18 ± 0.07	0.25 ± 0.05	**0.003**	0.386
CVI pararetina	0.17 ± 0.05	0.6 ± 1.57	**0.001**	0.816
CVI periretina	0.17 ± 0.03	0.22 ± 0.05	**0.002**	0.246
CVI whole image	0.2 ± 0.04	0.33 ± 0.38	**0.002**	0.913

CVI, choroid vascularity index; RP, retinitis pigmentosa; BCVA, best-corrected visual acuity

The choriocapillaris layer was evaluated in this study. 31 choriocapillaris high-quality (no missing or reversal areas) images from SS-OCTA examination were selected. Among them, 29 eyes’ segmentation of the Bruch membrane and the lower choroid boundary were corrected manually. A newly revealed CC defect pattern was detected in our study with a preserved CC in a concentric (25/31, 80.7%) or lobular pattern (6/31, 19.3%). No difference in anatomic changes and functional changes was found between the two CC defect patterns (Supplement Table 3). Correlation analysis revealed that different degrees of CC defect were correlated with the following functional assessment: BCVA (*p* = 0.001, r = − 0.556), Humphrey index (mean deviation, MD, *p* < 0.001, r = − 0.673; PVF, *p* = 0.003, r = − 0.639; 10° mean sensitivity, 10° MS, *p* = 0.002, r = − 0.651) and microperimetry index (mean sensitivity, MS, *p* < 0.001, r = − 0.807). In regard to anatomic changes, the CC defect degree was correlated with the VAD of the DCP in the fovea (*p* = 0.049, r = − 0.381), the EZ length (*p* < 0.001, r = − 0.627) and the average CVI of the parafoveal region (*p* = 0.036, r = − 0.511).

The ordinary regression model revealed that the CC defect degree was associated with the choroidal vascularity index (CVI) of the perifovea (*p* = 0.002, 95% CI: − 102.14- -24.01), EZ length (*p* = 0.006, 95% CI: − 0.006- -0.001) and the VAD of the DCP in the fovea (*p* = 0.022, 95% CI: 0.038–0.478).

Preserved CC areas in concentric CC defect patterns were measured using ImageJ software (open resources on the Internet). The average area was 28.65 ± 12.50 mm^2^ (out of 144 mm^2^ for the whole image). The preserved CC area was negatively correlated with microperimetry MS (*p* = 0.005, r = − 0.449).

## Discussion

In this study, retinal and choroidal vascularity were evaluated, and several facts were ascertained: 1) defects were observed in the CC layer with a concentric or lobular pattern in different degrees; 2) the CVI, which reflects middle/large choroidal vascularity, was decreased in the perifoveal, pararetinal and periretinal regions in RP eyes; 3) choriocapillaris defects and CVI defects were correlated with each other; 4) the CC defect degree was associated with visual function and retinal structural changes, including EZ length and the VAD of the SCP in the foveal region; 5) no correlations were detected between the CVI and visual function or retinal structural changes.

Choroidal vascularity changes were evaluated using SS-OCTA in this study, which can reveal choriocapillaris changes under RPE. Previous studies have reported CC flow defects in the retina around laser scars, in the geographic atrophy or drusen area of dry AMD patients in Stargardt disease, etc. [3, 4, 14, 15] CC defects with bare inner or middle choroidal vessels were observed in these studies. Hagag A M et al. also reported CC flow voids using 8 mm × 8 mm SD-OCTA in patients with different genotypes [7]. Manabu Miyata et al. reported concentric choriocapillaris flow deficits with bare large choroidal vessels in RP using 12 mm × 12 mm SS-OCTA [6], which was consistent with our findings. In Miyata’s research, no relationship between preserved CC area and BCVA was found. In our study, CC defects were first graded according to the appearance of different choroidal vascular structures, and correlations between visual function and CC were assessed comprehensively using BCVA, Humphrey perimetry and microperimetry. We also measured the CC preserved field, which has a clear relationship with MS in microperimetry (central 12°).

The choroid vascularity index (CVI) is a useful measurement for evaluating the middle and large choroid vessels, especially in choroidal hyperfusion diseases such as central serous chorioretinopathy [2] as well as degenerative diseases such as Stargardt disease [16]. Several reports on RP have reported that CVI decreases in B-scans passing through the fovea [9, 17]. Choroidal flow in the central 2.4 mm × 2.4 mm area was also assessed by using laser speckle flowgraphy (LSFG) [18]. However, these studies did not evaluate the left peripheral region of the retina, which may have differences from the central region and more likely to be affected in RP. Our study used three-dimensional OCTA and yielded a clearer map of CVI decrease, which indicated that middle and large choroid defects also begin from the periretinal region.

The relationship between retinal and choroidal vascularity was assessed in this study. The association between EZ length and CC defect degree revealed that photoreceptor loss kept in consistence with choriocapillaris integrity. The VAD of the DCP in the fovea was also consistent with the CC defects. However, the CVI and VAD in the different regions were not correlated, which may indicate that the choroidal and retinal defects in RP do not progress synchronously.

Retinal vascularity was also evaluated in this research. Defects of SCP and DCP were detected in RP eyes compared with control eyes, and VAD of DCP in fovea region were found to associated with BCVA. Our results were found to be consistent with those of previous studies [7, 19–23].

There are several limitations in our research. First, the sample size was relatively small in this study, and subgroup analysis with respect to the different CC defect degrees could not be performed due to the limited number of cases (less than 10). Second, acquiring high-quality OCTA images from low-vision eyes was relatively difficult, and patients with low vision had poor fixation ability, which led to reversal or blurred images, especially in the perifoveal region. Third, the retinal region outside the 40° × 40° was not assessed since current OCTA technology is relatively limited; the missing information from more perifoveal regions may assist more in understanding RP-related changes, and stitched images of more than 12 mm on a side that can depict 50° or larger of the retinal region should be acquired in further studies. Fourth, genetic effects were not considered in this study. Fifth, the retinal and choroidal vascularity has been evaluated in different OCTA machines since the Optovue and VG-200 has different emphases; Optovue is a classic spectral-domain OCTA with excellent algorithm for retinal vascularity evaluation, while VG-200 as a swept-source OCTA has more accurate images for choroidal vascularity evaluation. To make the contrast more clear, we have set two groups of healthy controls underwent different OCTA examinations to evaluate the retinal and choroidal vascularity changes separately.

## Conclusion

In conclusion, the choroidal vascularity was widely defective in RP. Choriocapillaris and middle/large choroidal vascularity defects were correlated with each other. Visual function and retinal structural changes were found to be associated with choriocapillaris defects but not with middle/large choroidal vascular defects, which indicated that CC defects are expected to be a more sensible index for predicting RP progression.

## Supplementary information

**Additional file 1.**

## Data Availability

All data generated or analysed during this study are included in this published article.

## Abbreviations

CC: Choriocapillaris
RP: Retinitis pigmentosa
OCTA: Optical coherence tomography angiography
CVI: Choroidal vascularity index
BCVA: Best-corrected visual acuity
MD: Mean deviation
PVF: Preserved visual field
CI: Confidence interval
EZ: Ellipsoid zone
VAD: Vascular area density
DCP: Deep capillary plexus
SCP: Superficial capillary plexus
RPE: Retinal pigment epithelium
SS-OCTA: Swept-source OCT angiography
IOP: Intraocular pressure
VA: Visual acuity
10° MS: 10° mean sensitivity
CFT: Central foveal thickness
SFCT: Subfoveal choroidal thickness
QI: Quality index
SD: Standard deviation
Min: Minimum
Max: Maximum
Q1: Lower quartile
Q3: Upper quartile
FAZ: Fovea avasular zone
AMD: Age-related macular degeneration
SD-OCTA: Spectral-domain OCTA
AXL: Axial length
RE: Refractory error
DS: Diopter sphere
DC: Diopter column
T: Tempora
S: Superior
N: Nasal
I: Inferior
