# Supplementary material for: Choroidal vascular changes in retinitis pigmentosa patients detected by optical coherence tomography angiography
Source: BMC Ophthalmol. 2020 Sep 29;20:384. doi: 10.1186/s12886-020-01640-5 (PMC7523071; doi:10.1186/s12886-020-01640-5)
Supplement: Supplementary file 1 — Additional file 1. [file 12886_2020_1640_MOESM1_ESM.docx]

Supplement Materials

Supplement Table 1. FAZ index in RP and control group

|  | control | RP | p |
| --- | --- | --- | --- |
| FAZArea | 0.32±0.09 | 0.32±0.16 | 0.935 |
| Perimeter | 2.19±0.36 | 2.26±0.65 | 0.444 |
| AcircularityIndex | 1.1±0.04 | 1.16±0.08 | **<0.001** |

FAZ, fovea avascular zone

Supplement Table 2. Correlation anaylsis between CVI and VD in different regions

|  | p value | |
| --- | --- | --- |
|  | CVI vs VD of SCP | CVI vs VD of DCP |
| fovea | 0.085 | 0.418 |
| parafovea | 0.314 | 0.293 |
| perifovea | 0.778 | 0.252 |

CVI, choroid vascularity index; VD, vessel density; SCP, superficial capillaris plexus; DCP, deep capillaris plexus

Supplement Table 3. Univariate analysis of anatomic and functional changes between different CC defect pattern

| CC defect pattern | Age, y | Disease Duration, y | BCVA | humphery_MD, dB | humphery_PVF, ° | humphery_10°meanMD, dB | Microperimetry_MD, dB |
| --- | --- | --- | --- | --- | --- | --- | --- |
| concentric | 43.17±12.86 | 31±12.87 | 0.38±0.29 | -29.24±3.99 | 8.7±5.36 | 8.99±7.79 | 8.06±6.6 |
| lobular | 31±16.23 | 20±15.33 | 0.32±0.39 | -21.68±8.46 | 16.1±9.18 | 16.5±9.91 | 13.65±10.67 |
| *p* value | 0.091 | 0.073 | 0.48 | 0.083 | 0.146 | 0.098 | 0.195 |
|  | CFT, μm | SFCT, μm | EZ lengthum, μm | VD in SCP of fovea | VD in SCP of parafovea | VD in SCP of perifovea | VD in DCP of fovea |
| concentric | 170.21±73.89 | 254.92±99.78 | 1048.63±881.19 | 14.87±15.69 | 40.2±5.41 | 48.27±5.26 | 30.53±9.43 |
| lobular | 197.08±106.29 | 248.17±114.74 | 1036.67±1162.87 | 20.33±14.94 | 41.18±4.76 | 47.5±2.7 | 31.68±10.22 |
| *p* value | 0.611 | 0.899 | 0.871 | 0.268 | 0.726 | 0.816 | 0.641 |
|  | VD in DCP of parafovea | VD in DCP of perifovea | CVI fovea | CVI parafovea | CVI perifovea | CVI pararetina | CVI periretina |
| concentric | 46.83±6.43 | 44.15±5.94 | 0.29±0.08 | 0.25±0.04 | 0.18±0.08 | 0.17±0.06 | 0.17±0.03 |
| lobular | 46.42±4.94 | 43.92±6.12 | 0.26±0.07 | 0.24±0.05 | 0.16±0.02 | 0.17±0.02 | 0.15±0.03 |
| *p* value | 0.771 | 0.726 | 0.492 | 0.751 | 0.712 | 0.752 | 0.245 |
|  | CVI whole retina | CC defect degree |  |  |  |  |  |
|  | 0.2±0.04 | 3.17±1.31 |  |  |  |  |  |
|  | 0.19±0.02 | 2.33±1.51 |  |  |  |  |  |
|  | 0.598 | 0.244 |  |  |  |  |  |

CC, choriocapillaris; BCVA, best-corrected visual acuity; MD, mean deviation; PVF, preserved visual field; CFT, central fovea thickness; SFCT, subfovea choroidal thickness; EZ, ellipsoid zone; VD, vessel density; SCP, superficial capillaris plexus; DCP, deep capillaris plexus; CVI, choroid vascularity index
